# Domestication Impacts the Wheat-Associated Microbiota and the Rhizosphere Colonization by Seed- and Soil-Originated Microbiomes, Across Different Fields

**DOI:** 10.3389/fpls.2021.806915

**Published:** 2022-01-12

**Authors:** Yulduzkhon Abdullaeva, Stefan Ratering, Binoy Ambika Manirajan, David Rosado-Porto, Sylvia Schnell, Massimiliano Cardinale

**Affiliations:** ^1^Institute of Applied Microbiology, Justus-Liebig-University, Giessen, Germany; ^2^Department of Biological and Environmental Sciences and Technologies – DiSTeBA, University of Salento, Lecce, Italy

**Keywords:** seed microbiome, bulk soil, crop domestication, coevolution, rhizosphere

## Abstract

The seed-transmitted microorganisms and the microbiome of the soil in which the plant grows are major drivers of the rhizosphere microbiome, a crucial component of the plant holobiont. The seed-borne microbiome can be even coevolved with the host plant as a result of adaptation and vertical transmission over generations. The reduced genome diversity and crossing events during domestication might have influenced plant traits that are important for root colonization by seed-borne microbes and also rhizosphere recruitment of microbes from the bulk soil. However, the impact of the breeding on seed-transmitted microbiome composition and the plant ability of microbiome selection from the soil remain unknown. Here, we analyzed both endorhiza and rhizosphere microbiome of two couples of genetically related wild and cultivated wheat species (*Aegilops tauschii*/*Triticum aestivum* and *T. dicoccoides*/*T. durum*) grown in three locations, using 16S rRNA gene and ITS2 metabarcoding, to assess the relative contribution of seed-borne and soil-derived microbes to the assemblage of the rhizosphere microbiome. We found that more bacterial and fungal ASVs are transmitted from seed to the endosphere of all species compared with the rhizosphere, and these transmitted ASVs were species-specific regardless of location. Only in one location, more microbial seed transmission occurred also in the rhizosphere of *A. tauschii* compared with other species. Concerning soil-derived microbiome, the most distinct microbial genera occurred in the rhizosphere of *A. tauschii* compared with other species in all locations. The rhizosphere of genetically connected wheat species was enriched with similar taxa, differently between locations. Our results demonstrate that host plant criteria for soil bank’s and seed-originated microbiome recruitment depend on both plants’ genotype and availability of microorganisms in a particular environment. This study also provides indications of coevolution between the host plant and its associated microbiome resulting from the vertical transmission of seed-originated taxa.

## Introduction

Plant domestication significantly altered the plant’s physiological, morphological, and genetic characteristics. The targeted and non-targeted selection for specific quality traits results in reduced allelic diversity of domesticated crops (Doebley et al., 2006). However, there is limited knowledge on how the alterations of plant genotype during the domestication influenced the assembly process of the rhizosphere microbiome composition.

The microbiome inhabiting plant habitats or compartments are known to influence plant health by creating intricate relationships with the host and can play important roles in plant survival (Santos-Medellín et al., 2017). One of the most important microbial habitats for plant health is the rhizosphere (Mendes et al., 2011). The assembly process of the rhizosphere microbiome composition starts immediately after the seed is placed in the soil, and the seed microbiome, the plant genotype, and the soil microbiome cooperatively shape the rhizosphere microbiome composition (Tkacz et al., 2020a; Walsh et al., 2021). Adequate work demonstrated the role of soil (Berg and Smalla, 2009; Bulgarelli et al., 2012; Lundberg et al., 2012; Schlaeppi et al., 2014) and host plants (Bulgarelli et al., 2012; Edwards et al., 2015; Tkacz et al., 2020b) in determining the structure of the rhizosphere microbiota. However, the dynamics of the seed-transmitted microbiome and plant characteristics that regulate microbial assembly and maintenance remain to be elucidated.

The vertically transmitted seed endophytes play a significant role in plant health, especially in the early stages of plant development (Johnston-Monje et al., 2016). The colonization of the rhizosphere by seed endophytes might be dependent on the host plant genotype. For example, quantitative trait nucleotides located on plant chromosomes can regulate mycorrhizal rhizosphere colonization as found by Ganugi et al. (2021) in tetraploid wheat genotypes. Moreover, seeds serve as a microbiological habitat for dispersal and dissemination, and this coexistence with the host for several generations eventually leads to plant–microbe coevolution (Abdullaeva et al., 2021). The symbiotic and mutualistic connections of seed endophytes with their hosts have been previously observed (Johnston-Monje et al., 2016; Nissinen et al., 2019). Therefore, changes in plant morphology, physiology, gene diversity loss in favor of selected plant traits during domestication such as seed characteristics (hard, soft, and big) or root/shoot architecture (Pérez-Jaramillo et al., 2017; Roucou et al., 2018) can influence the seed endophyte assembly (Abdullaeva et al., 2021). It is possible that the composition or frequency of seed endophytes that can transmit to the rhizosphere might differ and also a result of plant traits that facilitate or induce their transmission.

Furthermore, the seed-transmitted microbiome varies depending on the soil in which the plant is grown (Johnston-Monje et al., 2016). The seed endophytes, in contrast, may alter the composition of rhizosphere microbiota as they are initial rhizosphere inhabitants which initiate mutualistic, antagonistic, and symbiotic interactions with other soil microorganisms (Rybakova et al., 2017). The strong effect of soil on the bacterial microbiome assembly of wheat seedlings (*T. aestivum*) was recently showed by characterizing and comparing the bacterial composition of seed and soil on seedling microbiome in a broad range of soils (Walsh et al., 2021). However, the contribution of the seed bacterial and fungal microbiota to the adult plant rhizosphere microbiome, and also their survival degree in the rhizosphere, was rarely studied in plant holobiont investigations, because it is difficult to trace the transmitted endophytes from seed to rhizosphere during plant development. We could indeed gain knowledge about the significance of seed-originated microbes in shaping the rhizosphere microbiome by glancing into the magnitude of their contribution to the rhizosphere microbiota and how they survive in the rhizosphere across a variety of soil or host systems.

The rhizosphere is densely colonized by a myriad of microorganisms as the result of a major release of organic compounds by the plant roots. The organic carbon-like low molecular weight organic acids produced by plants are diverse and can impact the diversity and structure of the rhizosphere microbiome. Through the release of specific secondary metabolites and signaling molecules, plants can selectively recruit different microorganisms from surrounding soil (Bressan et al., 2009; Cotton et al., 2019). This causes changes in microbial diversity and activities around and inside the roots and also significantly influences the formation of specific root-inhabiting microbial communities for different plant species or genotypes, even when they grow in the same soil (Ofek-Lalzar et al., 2016). Domestication of crop plants can affect root exudates by changes at gene expression and protein modification level (regulatory and/or protein modifications in specific genes, structural heterogeneity, transposons, or genome doubling), and in the expression of genes responsible for the modification of precursors of secondary metabolites (Ober, 2005; Jacoby et al., 2021). Polyploidy (gene duplication) leads to the expansion of the gene catalog occurring in higher plant evolution that might contribute to the diversification of secondary metabolites (Jacoby et al., 2021). The variable secondary metabolites might lead to increased microbiome diversity in the rhizosphere of modern cultivars as reported by Cardinale et al. (2015) in wild and domesticated lettuce rhizosphere. Furthermore, the soil type and physicochemical soil properties have a significant impact on the specificity of the rhizosphere effect. Plants do recruit microorganisms from the soil reservoir, which is likely to differ in composition depending on the soil type. The degree of soil impact on the rhizosphere microbiome is determined by the structure of the soil microbiome due to the variable microbiota of soil able to colonize plant organs (Bulgarelli et al., 2012). Indeed, studies showed that a host plant’s rhizosphere effect can differ from one soil type to another (Bulgarelli et al., 2012; Lundberg et al., 2012). However, the question of how the genetic changes in plants impact their ability of microbe selection into the rhizosphere from different soils is left unanswered.

In this study, we investigated the microbiome associated with seed, root endosphere, rhizosphere, bulk soil, and soil before sowing (“seedbed”) of wild and domesticated cereals; the latter, as most genetically modified crops, offer perfect scenarios to evaluate the effect of genetic, physiological, and morphological changes caused by domestication on the rhizosphere microbiome selection processes. The rhizosphere microbiome of wheat has been well investigated (Donn et al., 2015; Yin et al., 2017; Schlatter et al., 2019, 2020; Tkacz et al., 2020b; Zhang et al., 2020). Moreover, a substantial number of studies focused on the characterization of the microbiomes associated with plant seeds (Robinson et al., 2016; Rahman et al., 2018; Alibrandi et al., 2020; Kuźniar et al., 2020; Abdullaeva et al., 2021), roots (Kavamura et al., 2020; Rossmann et al., 2020; Zhou et al., 2020), and bulk soil and the rhizosphere (Cardinale et al., 2015; Mahoney et al., 2017; Fan et al., 2018; Schlatter et al., 2020) of cereals. However, most of them were conducted under controlled conditions (greenhouse or laboratory), where environmental variability is strictly controlled or at least very limited.

Whereas previous studies have outlined the establishment of rhizosphere microbial communities across plant species, locations, and agro or ecosystem management, the question of whether cereal domestication influences the assembly process of the rhizosphere and, if so, how this effect differs across natural environments have received far less attention. Here, we characterized the bacterial and fungal microbiota of different soil or plant habitats of four wheat species cultivated in different soils at three locations. Aims of this work were to (1) compare diversity and composition of the bacterial and fungal microbiota in different plant habitats across three different locations, (2) assess the impact of plant domestication on the seed-transmitted microbiome and their relative contribution to the endosphere and rhizosphere microbiota, and (3) unravel the effects of changes in plant genotype during domestication and soil or environment on rhizosphere microbiome recruitment. We looked at how the structure of the rhizosphere microbiome of four wheat species shifted as a result of the interaction between plant species and the environment (location), focusing on the factors affecting the extent of rhizosphere colonization by soil- and seed-derived microbes.

We hypothesized that the relative contribution of the seed-transmitted microbiome to the rhizosphere microbiota of wild cereals will be higher than that of modern cereals. Comparison of seed-borne rhizosphere microbiome of different wheat species that grown in different locations allows us to observe coevolution patterns. We further hypothesized that the enriched bacterial and fungal microbiome in the rhizosphere from the soil will be more diverse in wild relatives than modern species as wild plants are genetically more diverse than modern plants. The outcomes of this study enhance our understanding of how the plant microbiome assembles and thus how the rhizosphere microbiome can be managed and/or manipulated to promote plant growth and health in sustainable agriculture.

## Materials and Methods

### Plant Material

Viable seeds of cereal species, *Triticum aestivum* L. ssp. *aestivum* (hereafter *“T. aestivum”*), *Triticum durum* Desf. ssp. *durum* (hereafter “*T. durum”*), and their corresponding wild ancestors, *Aegilops tauschii* Coss. ssp. *tauschii* (hereafter *“A. tauschii”*), and *Triticum dicoccoides* Schweinf. ssp. *dicoccoides* (hereafter *“T. dicoccoides”*), respectively, were obtained from the Leibniz Institute of Plant Genetics and Crop Plant Research (IPK), Germany. All seeds were propagated already for several years at the IPK, and collecting and storing were performed under the same conditions at the IPK. Once arrived at our laboratory, the seeds were stored in paper bags at 4°C until sowing.

### Experiment Design

Wheat and soil-associated microbiota was evaluated under field conditions, during the season 2018–2019. The experiment was set up with a randomized complete block design (three blocks: a, b, and c) at three research stations of the Justus-Liebig University of Gießen, Germany: Groß Gerau (GG), Weilburger Grenze (WG), and Rauischholzhausen (RH) (Supplementary Table 1). Wheat species, *A. tauschii*, *T. aestivum*, *T. dicoccoides*, and *T. durum* (Supplementary Table 2), were planted in each of three blocks in separate rows, randomly arranged to account for minor variations in soil and environmental conditions at small distance scale (Supplementary Table 1). Prior to sowing, seeds were carefully shelled, cleaned, surface-sterilized in 2.5% sodium hypochlorite for one min, and presoaked in water under sterile conditions for 24 h.

### Harvesting of Rhizosphere, Bulk Soil, Root, and Seedbed Samples

Seedbed samples were collected in triplicate from each location before sowing, to determine the primary soil microbial composition. At the plant flowering stage (May to July 2019), root, rhizosphere, and bulk soil samples were collected from all locations to study the microbiota around the plant root system. Plants were manually pulled out, carefully shaken to remove loosely attached soil, cut at the root–shoot boundary, and then placed into plastic bags. Bulk soil samples were collected from soil at the depth of rooting that was not closely adhering to the root. Collected samples were placed in cool boxes and transported to the laboratory, roots were further gently shaken, and then, the soil adhering to the roots was collected using a sterile scalp and sieved (2 mm) with a sterile sieve. Clean roots were placed into separate sterile 50-ml screw-cap tubes. Bulk soil and seedbed samples were also sieved and placed in sterile screw-cap tubes. All samples were frozen at −20°C until DNA extraction.

### Soil Analysis

Soil dry weight, water content, NH_4_^+^, NO_3_^–^, C_*t*_, N_*t*_, S_*t*_, and C:N ratio of both rhizosphere and bulk soils (two replicates each) were analyzed. NH_4_^+^, NO_3_^–^, and total C, N, S concentrations were measured on air-dried and 2-mm sieved samples. Approximately two g of each sample was finely ground using a RETSCH MM 400 Mixer Mill (Retsch GmbH, Haan, Germany) before total C, S, and N analysis using UNICUBE elemental analyzer (Elementar Analysensysteme GmbH, Langenselbold, Germany). Ammonia was determined using the method of Kandeler and Gerber (1988), after extraction with KCl. Nitrate was extracted from the soil as described by Cardinale et al. (2020) and measured with the ion chromatography method (Bak et al., 1991).

### DNA Extraction

Total DNA was extracted from the three samples of each set of the root (36), rhizosphere (36), bulk soil (36), and seedbed samples (9), using the PowerSoil^®^ DNA Isolation Kit (Qiagen, United States).

Before the genomic DNA extraction from roots, these were rinsed several times with sterile water until no further cloudiness was observed in the washing water. Washed roots were then treated with 2.5% sodium hypochlorite for 5 min. The samples were drained and rinsed with autoclaved, deionized water and then incubated in 70% ethanol for 2 min. The ethanol was removed, and samples were rinsed three times with autoclaved, deionized water. Roots were then crushed using sterile pestle and mortar in liquid nitrogen. Grounded roots were decanted into a 2-ml screw-cap tube that contains 200 μl of sterile glass beads and then frozen for later DNA extraction. The DNA was isolated from 300–500 mg of grounded samples as described by Abdullaeva et al. (2021).

Sequences of bacterial endophytes of seeds were taken from the previous study (Abdullaeva et al., 2021) (accession number: PRJEB36663), and the DNA already available from the previous study was used in this study to determine the fungal diversity by amplification of the ITS2 region. The seed pool used in the study of Abdullaeva et al. (2021) was the same used in this work for obtaining the field plants that are investigated. All extracted genomic DNAs were quantified by Nano-Drop™ 2000 Spectrophotometer (Peqlab, Erlangen, Germany) and then stored at –20°C until further analysis.

### Amplicon Library Preparation and Ion Torrent Sequencing

The V4-V5 regions of the 16S rRNA gene and the ribosomal internal transcribed spacer 2 (ITS2) from the rhizosphere, bulk soil, root, and seedbed were PCR-amplified to characterize the bacterial and fungal microbiota, respectively. The 16S RNA gene was amplified with the primer pair 520 F (5′-AYTGGGYDTAAAGNG-3′) (Claesson et al., 2009) and 907 R (5′-CCGTCAATTCMTTTRAGTTT-3′) (Engelbrektson et al., 2010) in combination with peptide nucleic acid (PNA) clumps, and purified, as described by Abdullaeva et al. (2021). The primer pair for the fungal ITS2 regions was ITS3 KYO2 forward (5′-GATGAAGAACGYAGYRAA-3′) and ITS4 reverse (5′-TCCTCCGCTTATTGATATGC-3′); amplification and purification were performed as described by Ambika Manirajan et al. (2018).

Polymerase chain reaction products were pooled in equimolar concentrations and used for emulsion PCR with Ion One Touch 2 (Ion PGM Hi-Q View OT2 kit, Life Technologies, Carlsbad, CA, United States). The quality of the final product was assessed using Ion Sphere Quality Control Kit (Life Technologies, Carlsbad, CA, United States) and loaded on a 314 or 318 chip for sequencing with an Ion PGM sequencer (Life Technologies, Carlsbad, CA, United States).

### Bioinformatic Analysis of the 16S rRNA and Internal Transcribed Spacer Amplicons

The raw 16S rRNA gene and ITS sequences were processed using the bioinformatic pipeline QIIME2 (version 2020.6) (Bolyen et al., 2019). Fungal ITS and 16S rRNA gene sequences were demultiplexed using Qiime2 *cutadapt* plugin (Martin, 2011). The ITS were then trimmed with ITS express (Rivers et al., 2018) deleting the flanking regions of the rRNA genes to leave only the ITS2 region. The QIIME2 plugin DADA2 was used for quality control, filtering, chimera identification, deionizing, clustering of the sequences to amplicon sequence variation ASV (99% sequence similarity), and producing the feature table. In the DADA2 step, 16S rRNA gene sequences were cut at position 320 bp and the first 15 bp were deleted. ITS2 sequences were already cut in the ITS express step, but to avoid a large number of ASVs because of the high length variability of the ITS region, the sequences were cut at position 150 bp (Rivers et al., 2018). Sequences were assigned to taxonomy with the QIIME2 plugin feature classifier (Bokulich et al., 2018) by pretrained naive Bayes classifiers (Pedregosa et al., 2011) trained on the SILVA 138 database (Quast et al., 2013) for the 16S rRNA gene sequences and the UNITE (v8.2) database (Kõljalg et al., 2013) for fungal ITS sequences. Thereafter, amplicon sequence variants (ASVs) that were identified as plastids or mitochondria were removed from the 16S rRNA gene sequences. The raw sequences were submitted to NCBI database^1^ under the project number (PRJNA773663).

### Statistical Analysis

Statistical analyses were performed in R-Studio (RStudio PBC, Boston, MA, United States) with R v.4.0.3 (R Core Team, 2020), using the ASV table generated from QIIME2 and were analyzed using the “phyloseq” package (McMurdie and Holmes, 2013).

Alpha-diversity was estimated using observed richness, Simpson, and Shannon diversity measures, using the mean value from ASV tables rarified to even depth. Significant differences between diversity indices among species, sample source, locations, and cultivation form (wild vs. cultivated) were determined using the Kruskal–Wallis rank-sum test.

Since the number of DNA sequence reads is restricted by the ability of the sequencing machinery, microbiome datasets that created by high-throughput sequencing are compositional (Gloor et al., 2016; Tsilimigras and Fodor, 2016). Instead of using regular counts and rarefying, we used a center log-ratio transformation (CLR) to evaluate the microbial composition of our datasets. Beta-diversity was assessed using a distance matrix based on Aitchison distance (Euclidian distance between samples) and variance-based compositional principal component analysis (PCA) plots (Aitchison, 1986; Aitchison and Greenacre, 2002). Significant differences in microbiota composition between groups and experimental factors were detected by permutational multivariate analysis of variance (ADONIS) (Anderson, 2001) using the vegan R package (Oksanen et al., 2017).

We conducted a multivariate homogeneity of group dispersion test to examine among community similarities between species, sample sources, and locations.

Constrained (canonical) ordination analysis was performed using RDA method to observe variation in the microbial communities between plant compartments, locations, and wheat cultivars by the environmental variables using RStudio with the rhizosphere and bulk soil data.

### The Relative Proportion of Seed-Transmitted Bacterial and Fungal Amplicon Sequence Variant Calculation

We used ASV counts for the identification of seed-transmitted microbiome proportion to the endorhiza and rhizosphere. The ASV counts for each replicate were manually related to the total seed ASV counts using excel, and the median of the relative proportion of replicates was used for graphical analysis. The seed-transmitted genera which were also found in seedbeds have not been considered seed-transmitted. Analysis of variance (ANOVA) was performed to identify the significant differences among the relative proportion of seed-transmitted microbiomes of wheat species and between locations using RStudio, followed by Tukey’s test to test significant differences or similarities between the specific groups.

### Differential Abundance Analysis Between Species and Locations

For the analysis of differential abundance between rhizosphere and bulk soil microbiome, the genera belonging to the core microbiome were used. The core microbiome of the rhizosphere, root, bulk soil, seed, and seedbed samples of each wheat species was identified using the “microbiome” package of RStudio by transforming their counts into relative abundances. Due to the high number of bacterial ASVs as compared to the fungi, 85% predominant bacteria genera and 75% fungal genera were used as threshold for the core grouping. These values are reasonable for calculating the core at genus level (Shade and Handelsman, 2012), and the assessment was made separately for each species in each location.

The differential abundance test was performed using *ALDE*x*2* (Fernandes et al., 2013) method to find microbial taxa with significant differential abundances between rhizosphere and bulk soil of each species which enable us to observe the bacterial and fungal microbiota enriched in the rhizosphere of genetically related groups (*A. tauschii*/*T. aestivum* and *T. dicoccoides*/*T. durum*).

For the evaluation of which genera are significantly enriched in the different treatments, the absolute ALDEx effect size (>1 and <−1) was used. For the graphical presentation, only enriched genera in the rhizosphere (>1) were used.

Furthermore, the compositional difference between the rhizosphere microbiome of wheat species that were grown in the same site (randomly selected) was tested using *ALDE*x*2* approach.

## Results

### 16S Amplicon Sequencing Results and Taxonomic Classification

The 16S rRNA gene amplicon sequencing yielded 2,690,188 high-quality, non-chimeric sequences across rhizosphere (631,004 sequences), bulk soil (661,618 sequences), root (1,076,002 sequences), and seedbed (321,564 sequences). Bacterial seed sequencing data that previously reported (Abdullaeva et al., 2021) and a partial of the bacterial seed sequences (24,204 sequences from seed accessions AE 220, TRI 368, TRI 18524, and TRI 10715) were used in this study. Two samples of *T. aestivum* from the rhizosphere dataset were removed because of low sequencing quality and number. We identified 27612 bacterial ASVs from 119 samples in total (34 rhizosphere, 36 bulk soil, 36 root, 9 seedbed, and 4 seed samples).

### Internal Transcribed Spacer Amplicon Sequencing Results

Sequencing of ITS amplicon library resulted in a total of 904,416 high-quality, non-chimeric sequences across rhizosphere (157,279 sequences), bulk soil (322,386 sequences), root (231,881 sequences), seedbed (176,963 sequences), and seed (15,907 sequences) samples. Two samples of *T. aestivum* from the rhizosphere, one sample of *T. dicoccoides* from the root datasets and one seedbed sample from Rauischholzhausen, were removed because of low sequencing quality and number. We identified 3,136 fungal ASVs from 117 samples in total (34 rhizosphere, 36 bulk soil, 35 root, 8 seedbed, and 4 seed samples).

#### Microbial Richness and Diversity of the Different Plant and Soil Compartments of Wheat Species

The alpha-diversity indices of bacterial and fungal ASVs were separately tested for significance of the factors location, plant habitat, cultivation form, and species (Supplementary Table 3). Moreover, the differences between habitats and species were determined for each location separately (Supplementary Table 3). The alpha-diversity indices of fungal rhizosphere or endorhiza and bulk soil microbiome significantly changed between locations in contrast to bacterial microbiomes of those habitats except for α-diversity indices of root endophytic bacterial microbiome (Supplementary Figure 1). Both fungal and bacterial microbiome α-diversities between habitats within locations were significantly different except for the bacterial microbiome in WG (Supplementary Table 3). Interestingly, the alpha-diversity of the bacterial microbiome in the bulk soil of four species was different in GG and RH (Supplementary Table 3). The alpha-diversities in the seedbed soil of the three locations of four wheat species were not different from each other (Supplementary Table 3). Cultivation forms of wheat species significantly affected the observed richness of fungal communities of bulk and root samples collected from RH.

#### Microbiota Differences Across Locations, Plant Compartments, Species, and Cultivation Form

**FIGURE 1:**
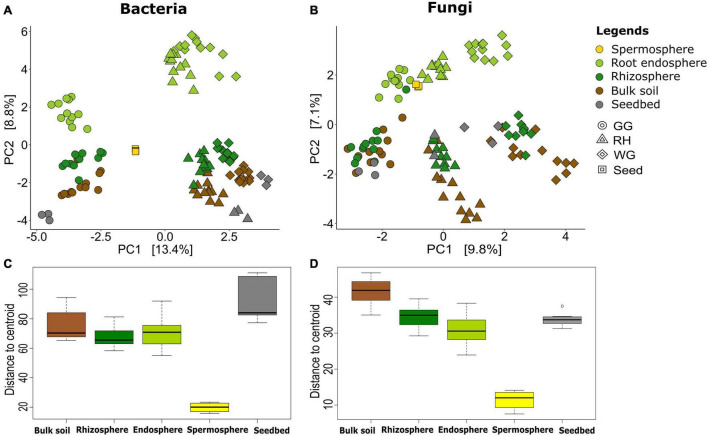
Similarity and variation among microbial communities within compartments. Unconstrained ordination based on Euclidian distance matrices of bacterial **(A)** and fungal **(B)** communities across rhizosphere, root, bulk soil, and seedbed samples collected from wheat species (*A. tauschii, T. aestivum. T. dicoccoides, and T. durum*) in three locations (GG, Groß-Gerau; WG, Weilburger Grenze; RH, Rauischholzhausen) and seeds obtained from the gene bank labeled as Seed. Euclidian distance calculated from the data transformed to the centered log-ratio. The colors of the dots denote the compartments of the samples: rhizosphere (forest green), root (light green), bulk soil (brown), seed (yellow), and seedbed (gray). The box plots represent the range of distances from the centroid based on Euclidian distance matrices of bacterial **(C)** and fungal **(D)** compositions. The black lines in the box plots correspond to median values, and the dots indicate outliers.

Aitchison distances visualized using PCA were used to investigate the beta-diversity. Both microbial communities were differentiated by the three locations; in particular, bacterial communities of GG were more different than other locations (Figure 1A), whereas fungal communities of all locations were equally dissimilar to each other (Figure 1B). The ordination results were further supported by permutational multivariate analysis of variance (ADONIS) based on Euclidian distance. ADONIS test results demonstrated that both bacterial (*R*^2^ = 0.144, *p* < 0.001) and fungal (*R*^2^ = 0.185, *p* < 0.001) communities were significantly differentiated by locations when considering all samples together (Figures 1A,B).

The bacterial communities were also separated by the five plant and soil compartments, and those in the seeds were found to retain the most distinguishable bacterial communities (Figure 1 and Supplementary Figure 2). The rhizosphere and root endosphere bacterial and fungal microbiome exhibited a community diversity that was more similar to each other than those of the other three compartments (Figures 1C,D and Supplementary Figure 2). However, the fungal communities were not well differentiated between soil compartments as compared to the bacterial microbiome (Figure 1B). The ordination results were further supported by permutational multivariate analysis of variance (ADONIS) based on Euclidian distance, which revealed significant separation of the bacterial (*R*^2^ = 0.119, *p* < 0.001) and fungal (*R*^2^ = 0.140, *p* < 0.001) communities by compartments, when considering all samples together (Figures 1A,B).

**FIGURE 2:**
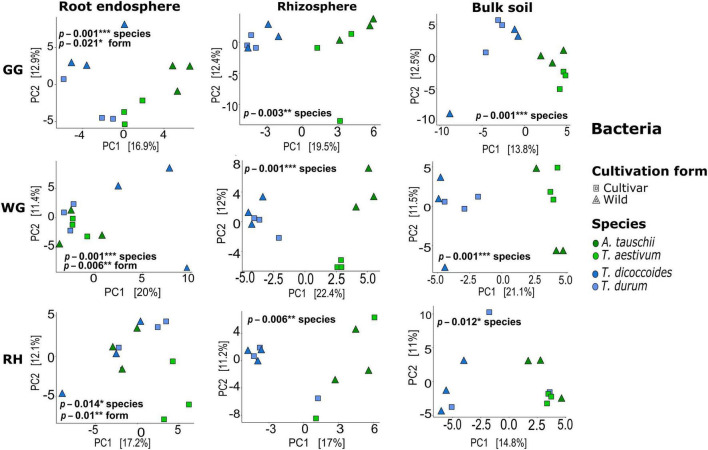
Bacterial beta-diversity in different compartments and locations. Unconstrained ordination based on Euclidian distance matrices of bacterial communities across the root, rhizosphere, and bulk soil samples collected from wild and domesticated wheat species (*A. tauschii*, *T. aestivum*, *T. dicoccoides*, and *T. durum*) in three locations (GG, Groß-Gerau; WG, Weilburger Grenze; RH, Rauischholzhausen). Euclidian distances calculated from the data were transformed to the centered log-ratio.

**FIGURE 3:**
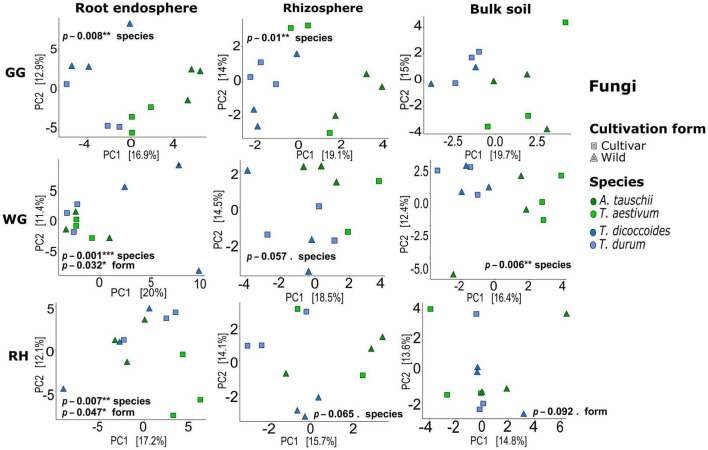
Fungal beta-diversity in different compartments and locations. Unconstrained ordination based on Euclidian distance matrices of bacterial communities across the root, rhizosphere, and bulk soil samples collected from wild and domesticated wheat species (*A. tauschii*, *T. aestivum*, *T. dicoccoides*, and *T. durum*) in three locations (GG, Groß-Gerau; WG, Weilburger Grenze; RH, Rauischholzhausen). Euclidian distances calculated from the data were transformed to the centered log-ratio.

Both bacterial and fungal communities of each plant compartment significantly differed across locations, and similarly, the communities of each location differed significantly across species and plant compartments (ADONIS, *p* < 0.05; Supplementary Table 4). Further tests were carried out to observe the effect of species and cultivation form factors within compartments and locations. Unconstrained ordination based on Euclidian distance matrices of bacterial and fungal microbiota showed that *T. aestivum* and its wild relative *A. tauschii* and also domesticated wheat *T. durum* and its wild relative *T. dicoccoides* were clustered together (Figures 2, 3). ADONIS results showed that the structure of both bacterial and fungal microbiota was significantly changed by the factor “cultivation form” in the root endosphere in all three locations except fungal microbiota in GG (Figures 2, 3). On the other hand, the factor “species” was significant for all plant compartments in each location, except than fungal microbiota of the bulk soil in GG (Figures 2, 3).

Homogeneity of variance of communities within the same compartments was examined by measuring the distance between the centroid and each sample of the group. Comparison of homogeneity of communities in plant compartments and locations showed significant dissimilarity (*p* = 0.001) among microbial communities of all sample sources (Figures 1C,D). The seed and rhizosphere bacterial and fungal communities exhibited the lowest dispersion, whereas bulk soil for fungi and seedbed for bacteria exhibited higher dispersion than other compartments (Figures 1C,D). Variations between the dispersion of seedbed bacterial communities and fungal communities were different.

#### Influence of Soil Characteristics on the Microbial Communities of the Root-Associated Microbiome of Wheat Species

Preliminary soil physicochemical characteristics and analysis of the collected rhizosphere and bulk soils provided a wide range of values across the samples (Table 1 and Supplementary Figure 3). ANOVA results showed that chemical soil properties (NO_3_^–^, *p* = 0.000035, NH_4_^+^, *p* = 0.0195, N, *p* = 0.0000009, C, *p* = 0.0632) with the exception of total carbon significantly differed between locations (*n* = 12) and also between plant compartments (rhizosphere and bulk soil; Supplementary Figure 3). Ammonia was significantly different between compartments only in GG (*p* = 0.0024), nitrate level was significantly different in WG (*p* = 0.0018) and RH (*p* = 0.0086), and nitrogen was only different in WG soil (*p* = 0.0049) (Supplementary Figure 3) between compartments.

**TABLE 1:** Geographical coordinates, soil type, and some important physical and chemical properties of studied field soils.

Location (abbreviation)	Geographical coordinates (elevation)	Soil type	pH	Sand (%)	Silt (%)	Clay (%)	Humus (%)	References
*Weilburger Grenze (WG)*	50°60′ N; 8°65′ E (158 m a.s.l.)	*Fluvic Gleyic Cambisol^a^*	6.0–6.4	6–15	40–58	36–48	2.20	Stumpf et al., 2019
*Rauischholzhausen (RH)*	56°76′ N; 8°88′ E (225 m a.s.l.)	*Haplic Luvisol^a^*	6.9–7.7	1.30–3.02	64.24	32	2	Macholdt et al., 2019; Wang et al., 2021
*Gross-Gerau (GG)*	49° 56′ N; 8° 30′ E (90.7 m a.s.l.)	*Arenosol* ^a^	6.5	85.2	9.6	5.2	1.1–1.5	Russo and Honermeier, 2017

*^a^Soil horizons were classified according to the World Reference Base for Soil Resource (WRB, 2014).*

Permutational ANOVA analysis on constrained axes used in ordination showed that the effect on the bacterial community composition of the rhizosphere and bulk soil samples was significantly different depending on the ammonia and moisture in the soil (Supplementary Figure 4 and Table 2). However, fungal communities changed by the nitrate in GG in both soil compartment and nitrogen content in RH only in the bulk soil (Table 2).

**TABLE 2:** Permutational ANOVA on constrained axes used in ordination (see Supplementary Figure 4).

Environmental variables	GG				WG				RH			
	Bacteria		Fungi		Bacteria		Fungi		Bacteria		Fungi	
	Rhizo	Bulk soil	Rhizo	Bulk soil	Rhizo	Bulk soil	Rhizo	Bulk soil	Rhizo	Bulk soil	Rhizo	Bulk soil
NH_4_^+^	0.033*	0.505	0.041*	0.737	0.224	0.001***	0.472	0.196	0.378	0.949	0.222	0.527
WC	0.116	0.047*	0.385	**0.075**	0.003**	0.018*	0.139	0.077	0.094	0.362	0.318	0.384
NO_3_^–^	0.123	0.613	0.040*	0.046*	0.300	0.336	0.276	0.948	0.12	0.727	0.638	0.476
C	0.102	0.181	0.567	0.317	0.198	0.383	0.585	0.373	0.121	0.567	0.748	0.580
N	0.495	0.573	0.422	0.229	0.176	0.349	0.336	0.562	0.714	0.59	0.418	0.005**
S	0.407	0.172	0.955	0.860	0.02*	0.357	0.600	0.546	0.071	0.421	0.696	0.182
C:N	0.272	0.198	0.604	0.090	0.294	0.357	0.847	0.717	0.358	0.767	0.141	0.649

RDA analysis was used for the bacterial and fungal community composition in the rhizosphere and the bulk soil of each location, and Euclidean distance was calculated for the environmental variables. The numbers indicate the permutational ANOVA significance (p) values, with significant values in bold and with asterisks, as indicated (<0.001 ‘^***^,’ <0.01 ‘^**^,’ <0.05 ‘*’). NH_4_^+^, ammonium; WC, water content; NO_3_^–^, nitrate; C, total carbon; N, total nitrogen; S, total sulfur; C:N, carbon–nitrogen ratio.

#### The Relative Proportion of Seed-Transmitted Endorhiza and Rhizosphere Bacterial Amplicon Sequence Variants

**FIGURE 4:**
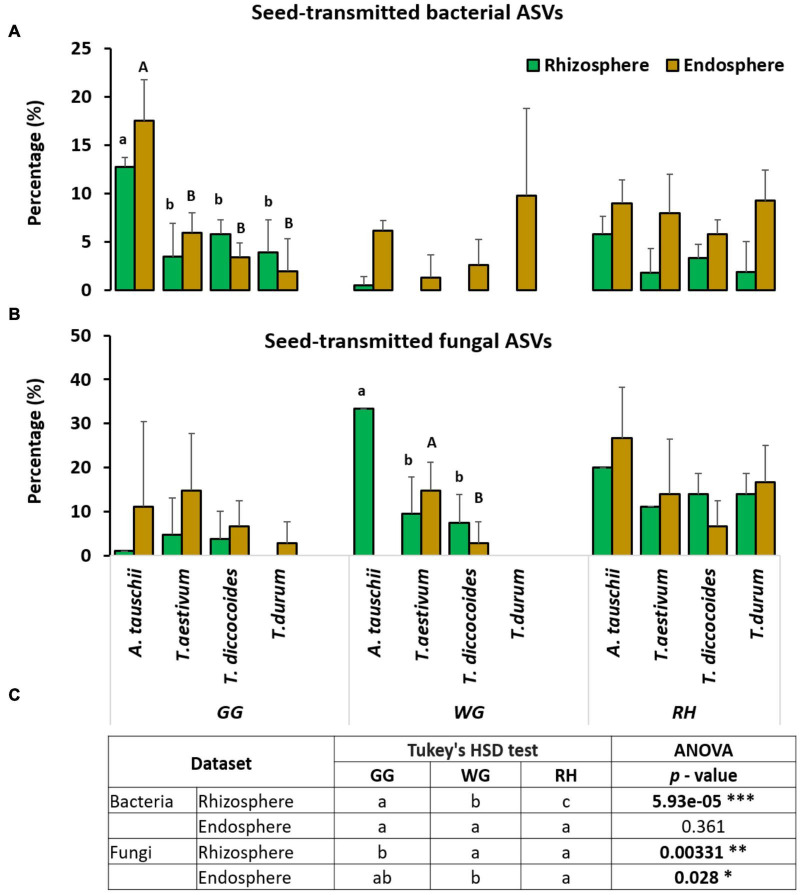
The relative proportion of seed-transmitted bacterial **(A)** and fungal **(B)** endorhiza and rhizosphere microbiome within each of the three locations (GG, WG and RH). Small letters show the significant differences (ANOVA, *p* < 0.05) between the relative proportion of seed-transmitted rhizosphere microbiome of wheat species. The capital letters show the significant difference between the relative proportion of seed-transmitted endorhiza microbiome of wheat species. **(C)** Differences between locations with respective p-values (*p < 0.05; **p < 0.01; ***p < 0.001).

In general, we observed a higher proportion of seed-derived bacterial and fungal microbiome in the endosphere compared with the rhizosphere. We also found a significantly higher proportion and also diversity of seed-derived microbiome in the endorhiza and rhizosphere of wild diploid *A. tauschii* than other wheat species (Supplementary Table 5). However, this pattern was observed in both bacteria and fungi, only in one location (bacteria in GG, fungi in WG) (Figures 4A,B). We also investigated the effect of location on seed transmission. The relative proportion of bacterial and fungal seed-transmitted rhizosphere microbiome was significantly influenced by location factor, whereas the effect of location has not been observed on the endorhiza bacterial microbiome (Figure 4C).

#### Seed-Originated Rhizosphere Microbiota

Amplicon sequence variants belonging to the genera *Verticiella*, *Chryseobacterium*, *Rhodococcus*, *Pseudomonas*, *Stenotrophomonas*, *Plantibacter*, *Methylobacterium*–*Methylorubrum*, *Luteibacter*, *Aeromicrobium*, *Cutibacterium*, *Allorhizobium–Neorhizobium–Pararhizobium–Rhizobium*, *Nocardioides*, *Massilia, Ulocladium*, and *Alternaria* were transmitted from seed to rhizosphere of *A. tauschii* (Supplementary Table 6).

*Pedobacter, Brevundimonas, Ulocladium, Stemphylium*, and *Alternaria* were transmitted from seed to rhizosphere of *T. aestivum* (Supplementary Table 6).

*Brevundimonas*, *Stenotrophomonas*, *Sphingomonas*, *Pseudomonas*, *Cutibacterium*, *Symbiobacterium*, *Pyrenophora*, *Ulocladium*, *Alternaria*, and *Neoascochyta* were transmitted from seed to rhizosphere of *T. dicoccoides* (Supplementary Table 6).

*Streptococcus*, *Ralstonia*, *Pseudomonas*, and *Alternaria* were transmitted from seed to rhizosphere of *T. durum* (Supplementary Table 6).

#### Seed-Originated Endorhiza Microbiota

Most of the genera found in the endorhiza were similar to the seed-transmitted rhizosphere microbes. *Rhodococcus*, *Enterobacteriaceae*, *Chryseobacterium*, *Verticiella*, *Pseudomonas*, *Stenotrophomonas*, *Allorhizobium–Neorhizobium–Pararhizobium–Rhizobium*, *Nocardioides*, *Luteibacter*, *Duganella*, *Comamonadaceae*, *Methylobacterium–Methylorubrum*, *Plantibacter*, *Cutibacterium*, *Aeromicrobium*, *Massilia*, unknown fungi, and *Alternaria* were transmitted from seed to endorhiza of *A. tauschii* (Supplementary Table 6).

*Brevundimonas*, *Pedobacter*, *Cutibacterium*, *Duganella*, *Massilia*, *Symbiobacterium*, unknown fungi, *Alternaria*, and *Stemphylium* were transmitted from seed to endorhiza of *T. aestivum* (Supplementary Table 6).

*Sphingomonas*, *Symbiobacterium*, *Cutibacterium*, *Stenotrophomonas*, *Pseudomonas*, *Neoascochyta*, *Alternaria*, and unknown fungi were transmitted from seed to endorhiza of *T. dicoccoides* (Supplementary Table 6).

*Pseudomonas*, *Streptococcus*, *Methylobacterium-Methylorubrum*, *Cutibacterium*, *Alternaria*, and unknown fungi were transmitted from seed to endorhiza of *T. durum* (Supplementary Table 6).

Among the seed-transmitted fungal genera, unknown fungi were transmitted from seed to endorhiza of all species and this is relevant for all three locations (Supplementary Table 6).

Some of the above-reported seed-transmitted genera were specific to particular wheat species and found at least in two locations. *Massilia, Methylobacterium*–*Methylorubrum, Pseudomonas, Plantibacter, Verticiella, Comamonadaceae, Allorhizobium–Neorhizobium–Pararhizobium–Rhizobium*, and *Stenotrophomonas* were specific to the endorhiza of *A. tauschii. Massilia* and *Methylobacterium*–*Methylorubrum* were specific to the rhizosphere of *A. tauschii* (Supplementary Table 6).

*Brevundimonas* was found specific to both endorhiza and rhizosphere of *T. aestivum, Pseudomonas, Streptococcus* to *T. durum*, and *Pseudomonas, Sphingomonas*, fungi *Pyrenophora*, and *Neoascochyta* were specific to *T. dicoccoides* (Supplementary Table 6).

Most of the fungi transmitted from seed to endorhiza and rhizosphere were specific to a particular location, such as, bacterial genera, *Symbiobacterium, Cutibacterium, and Pedobacter* and fungal genera *Ulocladium* and *Stemphylium* which were found in particular locations (Supplementary Table 6).

#### The Enriched Rhizosphere Microbiota as Compared to the Bulk Soil

**FIGURE 5:**
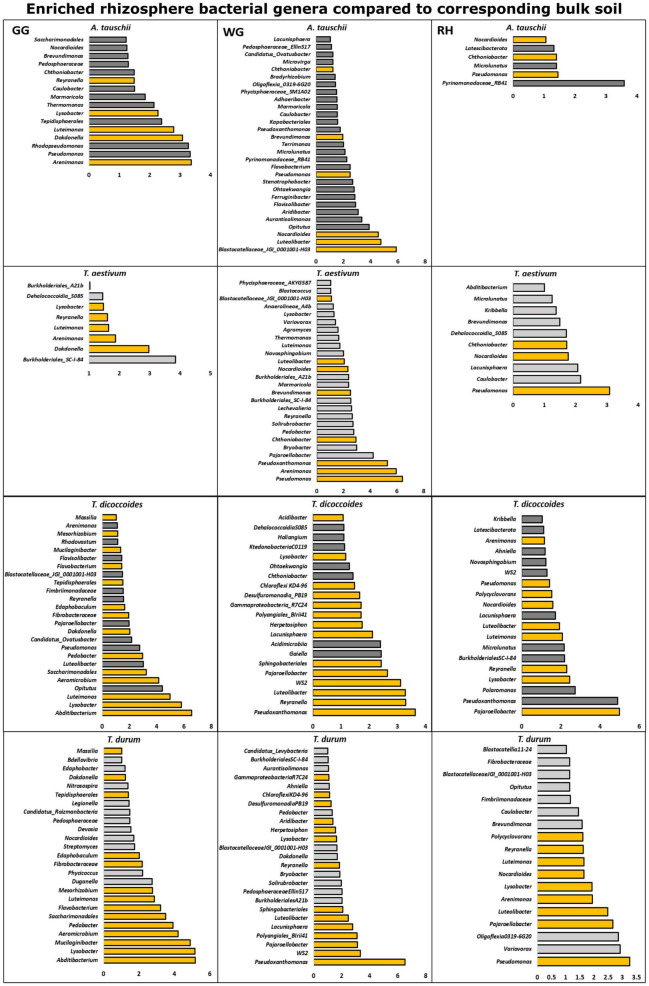
Bacterial genera that were found differently enriched in the rhizosphere of two genetically connected wheat species (wild *A. tauschii* vs. modern *T. aestivum;* wild *T. dicoccoides* vs. modern *T. durum*) were grown in three research fields (GG, Groß-Gerau; WG, Weilburger Grenze; RH, Rauischholzhausen) as compared to corresponding bulk soil. The differently abundant genera are considered as significant when absolute ALDEx affect size is bigger than 1. The dark gray color of bars (*n* = 3) indicates genera found in wild relative and light gray indicates modern wheat species. The orange color shows the genera found in genetically connected wheat species.

The differential abundance test showed that the rhizosphere of genetically connected couples of wheat species differently enriched bacterial and fungal genera from the bulk soil. The rhizosphere of *T. dicoccoides* and *T. durum* grown in the same location was found enriched with similar bacterial and fungal microbiome from the bulk soil (Figure 5), and the composition of the enriched microbiome was different in three locations.

**FIGURE 6:**
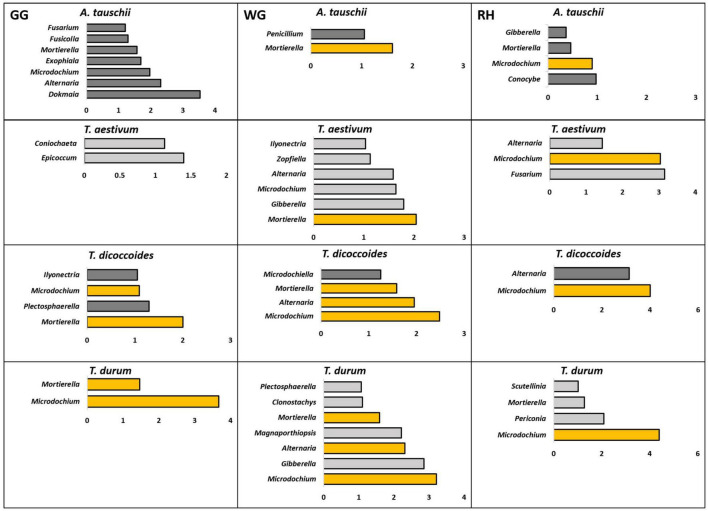
Fungal genera that were found differently enriched in the rhizosphere of two genetically connected wheat species (wild *A. tauschii* vs. modern *T. aestivum*; wild *T. dicoccoides* vs. modern *T. durum*) were grown in three research fields (GG, Groß-Gerau; WG, Weilburger Grenze; RH, Rauischholzhausen) as compared to corresponding bulk soil. The differently abundant genera are considered as significant when absolute ALDEx affect size is bigger than 1. The dark gray color of bars (*n* = 3) indicates genera found in wild relative and light gray indicates modern wheat species. The orange color shows the genera found in genetically connected wheat species.

The rhizosphere of modern *T. aestivum* and its wild ancestor *A. tauschii* was found enriched with a less similar bacterial microbiome than the other genetically related group from the corresponding bulk soil (Figure 5). Unlike the bacterial microbiome, fungal genera that were differentially enriched in the rhizosphere of wheat species were different from each other (Figure 6).

**FIGURE 7:**
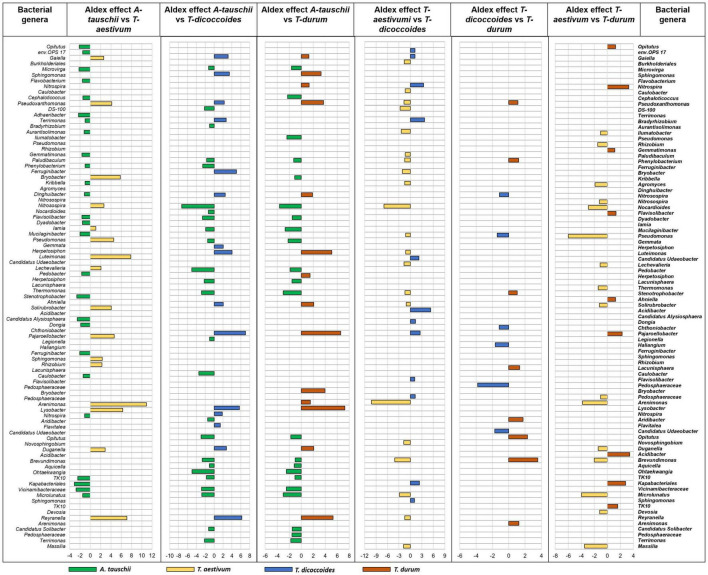
The rhizosphere bacterial microbiome assembly variation between wheat species (*A. tauschii*, *T. aestivum*, *T. dicoccoides*, and *T. durum*) grown in the same site (WG). The graph was created based on differential abundance analysis of core microbiome bacterial genera of rhizosphere soil. The significantly prevalent genera were identified by looking at ALDEx effect size table generated by ALDEx2. The differently abundant genera are considered as significant when absolute ALDEx affect size is bigger than 1 or lower than –1. More bars show higher differences and fewer bars explain more similarity between two wheat species.

Further analysis of differential abundance between rhizospheres of different wheat genotypes that were grown in the same site showed the more distinct bacterial rhizosphere microbiome assembly of wild *A. tauschii* from the other wheat genotypes (Figure 7). The most rhizosphere similarity observed between wild *T. dicoccoides* and modern *T. durum* (Figure 7) which are genetically connected. However, this result is not the same for the other genetically related couple (Figure 7). The second most similar rhizosphere microbiome was found between modern wheat species *T. aestivum* and *T. durum*. The rhizosphere of wild wheat species showed a more diverse however less abundant microbiome in contrast to modern wheat species.

**FIGURE 8:**
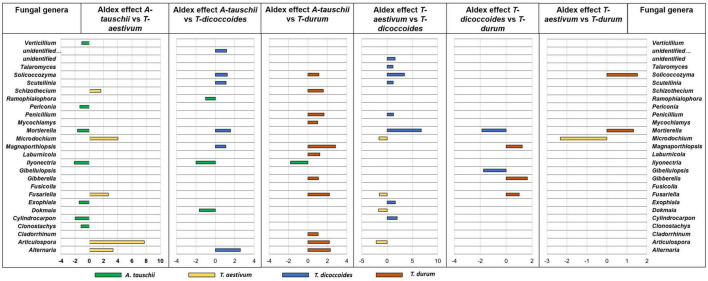
The rhizosphere fungal microbiome assembly variation between wheat species (*A. tauschii*, *T. aestivum*, T. *dicoccoides*, and *T. durum*) grown in the same site (WG). The graph was created on differential abundance analysis of core microbiome bacterial genera of rhizosphere soil. The significantly prevalent genera were identified by looking at ALDEx effect size table generated by ALDEx2. The differently abundant genera are considered as significant when absolute ALDEx affect size is bigger than 1 or lower than –1.

The differential abundance between rhizospheres of different wheat genotypes that were grown in the same site showed different fungal rhizosphere microbiome assembly between wild and modern wheat species. The most similar fungal rhizosphere microbiome was found between modern wheat species *T. durum* and *T. aestivum* (Figure 8). The more different fungal rhizosphere microbiome was found between wild and modern wheat species. However, genetically related wheat species wild, *T. dicoccoides*, and modern, *T. durum*, showed similar rhizosphere fungal microbiome assembly (Figure 8).

## Discussion

Plants have experienced considerable genetic, phenological, and physiological changes as a result of selection for certain quality attributes during domestication. This study used the 16S rRNA gene and ITS2 regions to determine the impact of plant domestication on main drivers of rhizosphere microbiome assembly, seed-transmitted and soil-originated, of four wheat species grown in different sites. The endorhiza and rhizosphere bacterial and fungal microbiomes were more comparable to one another than the seed microbiome (Figures 1C,D), suggesting that the majority of the endorhiza microbiome are originated from the rhizosphere which is consistent with previous studies (Bulgarelli et al., 2012; Leff et al., 2017) whereas seed has a unique environment which has no direct contact with the soil (Hardoim et al., 2012). We further found a significant effect of location (GG, WG, and RH) (Figures 1A,B) with more differentiation of bacterial communities between compartments than fungal microbiome. The results show the stronger effect of location on fungal microbiome than a plant which is similar to the findings of Bonito et al. (2014). Besides, the strong effect of plant genotype (*A. tauschii*, *T. aestivum*, *T. dicoccoides*, and *T. durum*) (Figures 2, 3) on the bacterial and fungal microbiome composition was observed. The findings are in line with the previously reported studies (Bouffaud et al., 2014; Walters et al., 2018; Schlatter et al., 2019). Over the last 20 years, the taxonomic composition of bacterial and fungal microbiomes in different plant habitats across different environments has been extensively studied. In this study, we wanted to place special emphasis on comprehending the impact of domestication on the seed-borne and soil-originated rhizosphere microbiome, to gain insight into the assembly process of the rhizosphere microbiome, one of the most crucial components of the plant holobiont.

Seed-transmitted endorhiza and rhizosphere microbiome of modern wheat species seem to be affected by domestication. We found a higher proportion of seed-borne microbes in the endorhiza and rhizosphere of diploid wild *A. tauschii* than modern wheat species. However, this is not true for another tetraploid wild wheat *T. dicoccoides*. Although, *T. dicoccoides* is wild wheat, its genome size, phenology, morphology are different than diploid *A. tauschii* and more similar to modern wheat species (Luo et al., 2007; Pont et al., 2019) since it has the same genome as *T. durum* and donated two genomes AA to bread wheat *T. aestivum* (Pont et al., 2019). Our results suggest that polyploidy events, even in older polyploid species, influence the transition (or survival) of seed endophytes to the endorhiza and rhizosphere. According to previous investigations, the genetic diversity was lost by 69% in hexaploid bread wheat and by 84% in tetraploid durum wheat during domestication (Haudry et al., 2007) as a result of polyploidy. Moreover, genome duplication also produces gene duplicates inside the same genome known as paralogs, which operate differently from the original gene due to a lack of selection pressure on one copy of the cloned gene (Scannell et al., 2007). The modified function of these redundant genes in the plant genome leads to a change in plant traits such as late flowering time, increased seed number as proved by Guo et al. (2014) in rapeseed, which might cause changes in its associated microbiome. A recent study also showed the effect of ploidy on the composition of the wheat bacterial root and rhizosphere microbiome in a greenhouse experiment; however, they did not observe the same results in a field experiment (Wipf and Coleman-Derr, 2021). Another similar study by Özkurt et al. (2020) showed that the seed-originated microbiome of roots and leaves of young seedlings was significantly less diverse and inconsistent in domesticated wheat species compared with the wild wheat species.

Furthermore, the higher relative proportion of seed-transmitted endosphere microbiome than rhizosphere microbiome indicates the coevolution of root endophytes with their host plant. The primary factors that lead to coevolution between wheat species and their endophytes are plant phylogeny (Yeoh et al., 2017) and niche adaptation over many years (Sessitsch et al., 2012; Özkurt et al., 2020). Yeoh et al. (2017) proved the role of plant phylogeny and its coadapted microbiome in shaping the root-associated microbiome of lycopods, ferns, gymnosperms, and angiosperms. Moreover, in our previous study, we also found phylogenetic congruence between seed endophytes and their host plants (Abdullaeva et al., 2021). One of the interesting findings of this study was the statistically significant difference in beta-diversity of both, bacterial and fungal microbiome which found in the endorhiza and not in the rhizosphere, of wild and modern wheat species across the two couples studied (Figures 2, 3). This suggests a microbe–host coevolution, which is expected to be more important in the endosphere. In fact, when seed endophytes colonize the rhizosphere, their proportion gets smaller due to the vast array of microbes attracted from the bulk soil and this potentially reduces the coevolution factor. This is likely the reason why we observed a reduced difference in between the rhizosphere microbiota of wild and domesticated wheat species with respect to the endosphere, where differences appear to be more pronounced.

Our experimental design allowed us to observe the effect of domestication on the seed-transmitted bacterial and fungal rhizosphere microbiome of wheat species in different locations. We found a higher relative proportion of seed-transmitted bacterial endosphere, rhizosphere microbiome of diploid wild wheat in GG, and seed-transmitted fungal rhizosphere microbiome in WG (Figure 4). The observed differences between locations (Figures 4A,B) agree with recent work by Walsh et al. (2021) where the variable proportion of seed endophytes to the wheat seedling microbiome was found between different soils. These authors also showed a strong effect of soil on seedling microbiome assembly where dominant microbes are transmitted from seed (Walsh et al., 2021). Moreover, Özkurt et al. (2020) observed the seed-transmitted microbiome of seedlings of cultivated and wild wheat species in two different soils and found a strong effect of soil on the seedling microbiome. The effect of location on the rhizosphere is commonly observed in microbiome studies as the rhizosphere directly contacts the soil. In our study, the proportion of seed-transmitted endorhiza fungi, not bacteria, significantly varied between locations (Figure 4C). Similarly, we found significant changes in the alpha-diversity of the only fungal endorhiza microbiome between locations (Supplementary Figure 1). These results suggest that soil origin or environment had a greater impact on the fungal population assemblage in the root endosphere and rhizosphere rather than host species. Our results are coherent with those of several recent studies, which also indicate the stronger effect of soil or environment in controlling the dynamics of microbial seed transmission (Bonito et al., 2014; Özkurt et al., 2020; Morales Moreira et al., 2021; Walsh et al., 2021).

The analyses of environmental variables on the rhizosphere microbiome showed that the bacterial and fungal species were significantly affected depending on the ammonium and nitrate content of soil (Supplementary Figure 4 and Table 2). Indeed, a low concentration of ammonium and nitrate was determined in the sandy soils of GG compared with loamy clay soils of WG and RH area (Supplementary Figure 3). These results indicate that the proportion of seed-transmitted microbiome varies depending on soil characteristics. Furthermore, the proportion of seed-transmitted rhizosphere microbiome of wild *A. tauschii* can be higher under lack of nitrogen source. Indeed, most of the seed-transmitted bacteria from seed to rhizosphere of *A. tauschii* were the plant growth-promoting bacteria with the ability to fix N_2_ and enhance mineralization. For example, *Chryseobacterium* carries nod gene *nifH* and its ability of nitrogen fixation was confirmed when inoculated with groundnut (Dhole et al., 2016). *Rhodococcus* harbors a *nodA* gene (Ampomah and Huss-Danell, 2011). *Methylobacterium–Methylorubrum* and *Allorhizobium–Neorhizobium–Pararhizobium–Rhizobium* genus belong to the phylogenetic rhizobial branch which functionally conserves nodulation genes (Sy et al., 2001; Renier et al., 2008). *Plantibacter* (Mayer et al., 2019), *Pseudomonas*, *Burkholderia*, and other non-rhizobial endophytic bacteria were found in nodules and aid in nitrogen fixation in particular stress conditions (Martínez-Hidalgo and Hirsch, 2017). Our findings suggest that modern wheat became less effective in making beneficial interactions with its associated microbes to cope with environmental stressors. As shown in a recent paper, there is a downward trend in making beneficial interactions in terms of N mineralization as wheat domesticated: diploid > tetraploid > hexaploid (Spor et al., 2020). Seed-transmitted microbiome-mediated microbial interaction leads to diverse rhizosphere microbiomes as we found in this study (Figure 7). However, these suggestions about potential N-related functions will need to be confirmed by future functional-based studies.

We found that the rhizosphere microbiome of *A. tauschii* is more different than other wheat species by comparing differently enriched genera between the rhizosphere microbiome of four wheat species (Figure 7). First, domestication-related changes in the plant genome, such as gene loss, genomic rearrangements, and gene duplications (Doebley et al., 2006; Pont et al., 2019), significantly influenced plant traits (Szoboszlay et al., 2015; Roucou et al., 2018; Spor et al., 2020) that shape microbiome in different habitats of plants. As such root exudate content, an important plant trait for assembly process of the rhizosphere as described by Mönchgesang et al. (2016) discovered strong variations in root exudate chemistry among *Arabidopsis* accessions depending on genetic characteristics. Moreover, *T. aestivum* gene diversity significantly reduced as a result of subsequent polyploidy events. Its D genome was found to conserved more trait *loci* than in subgenomes A and B (Berkman et al., 2011); however, it was also found that subgenome D can modify 42.8% of alternative splicing patterns (during gene expression, an alternative splicing process allows a single gene to code for numerous proteins) of subgenomes A and B (Yu et al., 2020), meaning that domestication at the hexaploid level had a greater effect on genetic modifications between subgenomes than same processes at the tetraploid level (Lv et al., 2021). This suggests that subsequent polyploidy events lead to the loss of more genetic information to recruit microorganisms from the bulk soil. These findings are in line with previous work in which the effect of plant domestication on the rhizosphere microbiome of different plant genetic groups of maize (*Zea* mays), and they found greater similarity of microbiome composition between the rhizosphere microbiome of inbred maize varieties and the teosinte than the hybrid lines (Brisson et al., 2019). Furthermore, plant specifically selects microbes from the bulk soil depending on its genotype. Tkacz et al. (2020b) found that the wheat lines crossed with *A. tauschii*, containing wild D genome, were highly colonized specifically by *Glomeromycetes* and Nematoda by testing several wheat species; wild (*A. tauschii*, *T. dicoccoides*), elite (*T. aestivum*, *T. durum*), and hybrid (SHW) wheat lines. Another study found sex plant qualitative traits in 94 winter wheat genotypes that are responsible for this symbiotic interaction with arbuscular mycorrhizal fungi (Lehnert et al., 2017).

Previous studies reported a significant difference between genomic (Haudry et al., 2007; Peleg et al., 2011), phenotypic diversity (Gioia et al., 2015), and rhizosphere microbiome (Spor et al., 2020; Özkurt et al., 2020) between wild *T. dicoccoides* and *T. durum*. In this study, we observed similar bacterial and fungal taxa enriched the rhizosphere of modern *T. durum* and wild relative *T. dicoccoides*. The geographical distribution of the wheat population might explain the similarity of microbiome recruitment of wild emmer with modern wheat species (Luo et al., 2007). Growing in a similar region, the environment can lead to the introgression of domesticated wheat genes into the wild wheat genome (Weide, 2015). Durum wheat was found closely related to progenitor species distributed in the eastern Mediterranean (Israel, Cyprus, Palestine, Greece, Syria Lebanon, Turkey, Jordan, and Egypt). The origin of *T. dicoccoides* (Israel) and *T. durum* (Greece) genotypes that were used in this study was from the same region (Supplementary Table 2). Furthermore, our previous studies showed strong genetic concordance (UPGMA dendrogram) among *T. dicoccoides*, *T. aestivum*, and *T. durum* than *A. tauschii* (Abdullaeva et al., 2021).

Furthermore, we found microbes specifically enriched under specific plants in a particular location (Figure 4). For instance, the rhizosphere microbiome composition of wheat species, specially cultivated *T. durum*, and its ancestor *T. dicoccoides* were similar in all three locations (Figure 4); however, the enriched genera were different in each location. These results also suggest that similar plants do recruit potential rhizosphere colonizing bacterial species available in the soil where they grow and may indicate a strong effect of the growing site on the rhizosphere bacterial assembly but modulated by the plant genotype. Results of constrained ordination analyses also showed a significant effect of growing site and its soil parameters on the rhizosphere microbiota composition (Supplementary Figure 4), and the results further supported by the differential abundance test showed that the genera were significantly affected by the factor location (Figure 4 and Supplementary Figures 5, 6). The rhizosphere bacterial and fungal microbiome abundance of the same plant (*A. tauschii*) that grown in three locations was also different (Supplementary Figures 5, 6). Results imply that soil microbes play a pivotal role in determining the rhizosphere microbiota composition, coherently with previously reported studies (Bulgarelli et al., 2012; Lundberg et al., 2012; Wagner et al., 2014).

## Conclusion

Our findings indicate that the seed-transmitted microbiome of endorhiza and rhizosphere is impacted by crop domestication. We showed polyploidy effect, by finding less relative seed-transformed microbiome in the endorhiza and rhizosphere of tetraploid and hexaploid wheat species, including wild emmer wheat *T. dicoccoides* than the diploid ancestor *A. tauschii*. We further showed the importance of the seed-transmitted microbiome in shaping the rhizosphere microbiome by identifying the members of these seed-borne microbes. Moreover, we showed the strong effect of the environment on the relative proportion of seed-transmitted microbiome and also rhizosphere microbial recruitment from the bulk soil. This study also provides some notable clues of coevolution between the host plant and its microbiome during domestication.

Assessing how the plant microbiome altered since plant domestication and how this effect varies across locations and plant species can help us to predict how the plant microbiome can be modified or manipulated to improve plant health and crop productivity.

## Data Availability Statement

The datasets presented in this study can be found in online repositories. The names of the repository/repositories and accession number(s) can be found below: https://www.ncbi.nlm.nih.gov/, PRJNA773663.

## Author Contributions

YA contributed to methodology, investigation, and writing – original draft. SR contributed to methodology, supervision, and writing – review and editing. BA and DR-P contributed to methodology and investigation. SS and MC contributed to conceptualization, resources, supervision and writing – review and editing. All authors contributed to the article and approved the submitted version.

## Conflict of Interest

The authors declare that the research was conducted in the absence of any commercial or financial relationships that could be construed as a potential conflict of interest.

## Publisher’s Note

All claims expressed in this article are solely those of the authors and do not necessarily represent those of their affiliated organizations, or those of the publisher, the editors and the reviewers. Any product that may be evaluated in this article, or claim that may be made by its manufacturer, is not guaranteed or endorsed by the publisher.

## Footnotes

1
www.ncbi.nlm.nih.gov/
