# Supplementary material for: Domestication Impacts the Wheat-Associated Microbiota and the Rhizosphere Colonization by Seed- and Soil-Originated Microbiomes, Across Different Fields
Source: Front Plant Sci. 2022 Jan 12;12:806915. doi: 10.3389/fpls.2021.806915 (PMC8789879; doi:10.3389/fpls.2021.806915)
Supplement: Supplementary file 1 [file Data_Sheet_1.PDF]

Supplementary material to:

Domestication impacts the wheat-associated microbiota and the rhizosphere colonization by seed- and soil-originated microbiomes, across different fields

Yulduzkhon Abdullaeva[1], Stefan Ratering[1], Binoy Ambika Manirajan[a#], David Rosado-Porto[1], Sylvia Schnell[1*] and Massimiliano Cardinale[1,2*]

[1]Institute of Applied Microbiology, Justus-Liebig-University, Giessen, Germany

[2]Department of Biological and Environmental Sciences and Technologies – DiSTeBA, University of Salento, Lecce, Italy

[#]Current affiliation: School of Biosciences, Mahatma Gandhi University, Priyadarsini Hills, Kottayam, Kerala, India

*Corresponding Authors:

Massimiliano Cardinale, Tel: +39-0832297078, E- mail:massimiliano.cardinale@unisalento.it

Sylvia Schnell, Tel.: +49- 641-9937350, E-mail: sylvia.schnell@umwelt.uni-giessen.de

- **Table S1.** Sampling design, sampling locations (GG-Groß-Gerau, WG-Weilburger Grenze, and RH- Rauischholzhausen) and frequency of sampling for different habitats.
- **Table S2.** Origin and biological status of the seed accessions used in this work.
- **Table S3**. Kruskal-Wallis rank-sum test results. Significant differences in microbial communities richness and diversity between groups and experimental factors within datasets were detected (available as excel file).
- **Table S4.** Permutational multivariate analysis of variance (ADONIS) test results. The numbers indicate the ADONIS statistic (r) value, with significance as indicated (< 0.001 '***', < 0.01 '**', < 0.05 '*', < 0.1 '.'). Significant differences in microbial communities composition between groups and experimental factors within datasets were detected.
- **Table S5.** Total counts of ASVs from four wheat seeds and ASVs that were transmitted wither to the root endosphere or to the rhizosphere. Note that seed ASVs shared with the respective seedbed in each location were removed from this calculation (which explains the different number of total ASVs of each species in the different locations).
- **Table S6.** Composition of seed-transmitted endorhiza and rhizosphere bacterial and fungal in the three locations (GG, WG, RH) (available as excel file).
- **Figure S1.** Observed richness and diversity (Shannon's index and Simpson indexes) of bacterial (A) and fungal (B) microbiota of different habitats (root endosphere, rhizosphere, bulk soil) between locations: yellow – Gross-Gerau (GG), green – Rauischholzhausen (RH), and brown – Weilburger Grenze (WG). Box plots show the range of variation in the median values (black lines in the middle), and the dots indicate outliers. Asterisks indicate significant differences between locations (*** = $p < 0.001$; ** = $p < 0.01$; * = $p < 0.05$).
- **Figure S2.** Bacterial (A) and fungal (B) taxa distribution in different compartments of wheat cultivars grown in three locations. Relative abundance of 95% predominant bacterial and fungal phyla and 99% predominant genera. Each bar represents 9 samples (3xGG, 3xWG, 3xRH).
- **Figure S3.** Soil properties. Ammonium (NH_4^+), nitrate (NO_3^-), nitrogen (N), and carbon (C) of rhizosphere and bulk soil (n=12). Soil samples collected from three locations Weilburger Grenze (WG), Groß-Gerau (GG), and Rauischholzhausen (RH). One-way ANOVA results. All parameters (NO_3^-, $p = 0.000035$, NH_4^+, $p = 0.0195$, N, $p = 0.0000009$, C, $p = 0.0632$) significantly differed between locations (n = 12). The bars represent the

mean values of 3 replicates of each wheat species, error bars show standard deviation. Significance values between rhizosphere and bulk soil in each location are represented on the right corner of each barplot.

- **Figure S4.** Constrained (canonical) ordination analyses. The effect of environmental variables on bacterial and fungal species in the rhizosphere and bulk soil samples of cereals.
- **Figure S5.** The bacterial structure variation between locations (GG-Groß-Gerau, WG-Weilburger Grenze, RH-Rauischholzhausen) based on differential abundance analysis of core microbiome bacterial genera of rhizosphere (*A. tauschii*). The significantly prevalent genera were identified by looking at aldex effect size. The differently abundant genera are considered as significant absolute aldex affect size bigger than 1 or lower than -1.
- **Figure S6.** The fungal structure variation between locations (GG-Groß-Gerau, WG-Weilburger Grenze, RH-Rauischholzhausen) based on differential abundance analysis of core microbiome bacterial genera of rhizosphere (*A. tauschii*). The significantly prevalent genera were identified by looking at aldex effect size. The differently abundant genera are considered as significant absolute aldex affect size bigger than 1 or lower than -1.

Table S1 Sampling design, sampling locations (GG-Groß-Gerau, WG-Weilburger Grenze, and RH- Rauischholzhausen) and frequency of sampling for different habitats.

Species	Location	Accession number	Roots	Rhizosphere	Bulk soil	Seedbed
T. diccocoides	GG	TRI 18524	3	3	3	3
	WG	TRI 18524	3	3	3	3
	RH	TRI 18524	3	3	3	3
T. durum	GG	TRI 10715	3	3	3	
	WG	TRI 10715	3	3	3	
	RH	TRI 10715	3	3	3	
T. aestivum	GG	TRI 368	3	3	3	
	WG	TRI 368	3	3	3	
	RH	TRI 368	3	3	3	
A. tauschii	GG	AE 220	3	3	3	
	WG	AE 220	3	3	3	
	RH	AE 220	3	3	3	

Table S2. Origin and biological status of the seed accessions used in this work.

Scientific name of the accession	Biological status	IPK* Accession number
Aegilops tauschii Coss. subsp. *tauschii* var. *meyeri* (Griseb.) Tzvelev	wild	AE 220
Triticum aestivum L. var. *aestivum*	cultivar	TRI 368
*Triticum dicoccoides (*Körn. ex Asch. & Graebn.) Schweinf. convar. *dicoccoides* var.*dicoccoides*	wild	TRI 18524
Triticum durum Desf. subsp. *durum* convar. *durum* subconvar. *durum* var. *affine* Körn.	cultivar	TRI 10715

* Leibniz Institute of Plant Genetics and Crop Plant Research (IPK)

Table S3. Kruskal-Wallis rank-sum test results. Significant differences in microbial communities richness and diversity between groups and experimental factors within datasets were detected (available as excel file)

Table S4. Permutational multivariate analysis of variance (ADONIS) test results. The numbers indicate the ADONIS statistic (r) value, with significance as indicated (< 0.001 '***', < 0.01 '**', < 0.05 '*', < 0.1 '.'). Significant differences in microbial communities composition between groups and experimental factors within datasets were detected. Locations: GG-Groß-Gerau, WG-Weilburger Grenze, RH- Rauischholzhausen.

**Dataset**	**Factor**	**Bacteria**			**Fungi**		
		**Df**	$\mathbf{R^2}$	***P***	**Df**	$\mathbf{R^2}$	***P***
**Rhizosphere**	species	3	0.10017	0.173	3	0.08019	0.741
	location	2	0.29548	**0.001 *** **	2	0.26305	**0.001 *** **
	form	1	0.02379	0.75	1	0.02604	0.632
**Bulk soil**	species	3	0.08598	0.413	3	0.08571	0.552
	location	2	0.28887	**0.001 *** **	2	0.2126	**0.001 *** **
	form	1	0.02113	0.942	1	0.02762	0.542
**Root endosphere**	species	3	0.08722	0.385	3	0.08877	0.313
	location	2	0.31587	**0.001 *** **	2	0.2216	**0.001 *** **
	form	1	0.03324	0.22	1	0.02802	0.435
**Seedbed**	location	2	0.55212	**0.005 ** **	2	0.47079	**0.008 ** **
**Separately tested by location**							
**GG**	species	3	0.11019	**0.012 * **	3	0.10794	**0.019 * **
	form	1	0.02927	0.334	1	0.03086	0.288
	plant compartment	2	0.17392	**0.001 *** **	2	0.1697	**0.001 *** **
**WG**	species	3	0.12302	**0.012 * **	3	0.10233	**0.065 .**
	form	1	0.03252	0.201	1	0.03223	0.203
	plant compartment	2	0.24235	**0.001 *** **	2	0.19663	**0.001 *** **
**RH**	species	3	0.0962	0.213	3	0.0807	0.68
	form	1	0.02933	0.36	1	0.03063	0.257
	plant compartment	2	0.22413	**0.001 *** **	2	0.18447	**0.001 *** **

Table S5. Total counts of ASVs from four wheat seeds and ASVs that were transmitted wither to the root endosphere or to the rhizosphere. Note that seed ASVs shared with the respective seedbed in each location were removed from this calculation (which explains the different number of total ASVs of each species in the different locations.

		Location											
		Groß-Gerau (GG)				Weilburger Grenze (WG)				Rauischholzhausen (RH)			
	**Species**	*A. tauschii*	*T. aestivum*	*T. dicoccoides*	*T. durum*	*A. tauschii*	*T. aestivum*	*T. dicoccoides*	*T. durum*	*A. tauschii*	*T. aestivum*	*T. dicoccoides*	*T. durum*
**Rhizosphere**													
Total seed ASVs		60	29	40	17	64	29	40	18	63	28	40	18
Transmitted ASVs		11	2	4	2	1	0	0	0	5	2	4	1
**Endosphere**													
Total seed ASVs		59	28	39	17	54	25	38	17	63	29	40	18
Transmitted ASVs		15	3	2	1	6	1	2	3	9	5	6	3

Table S6 Composition of seed-transmitted endorhiza and rhizosphere bacterial and fungal in the three locations (GG, WG, RH) (available as excel file).

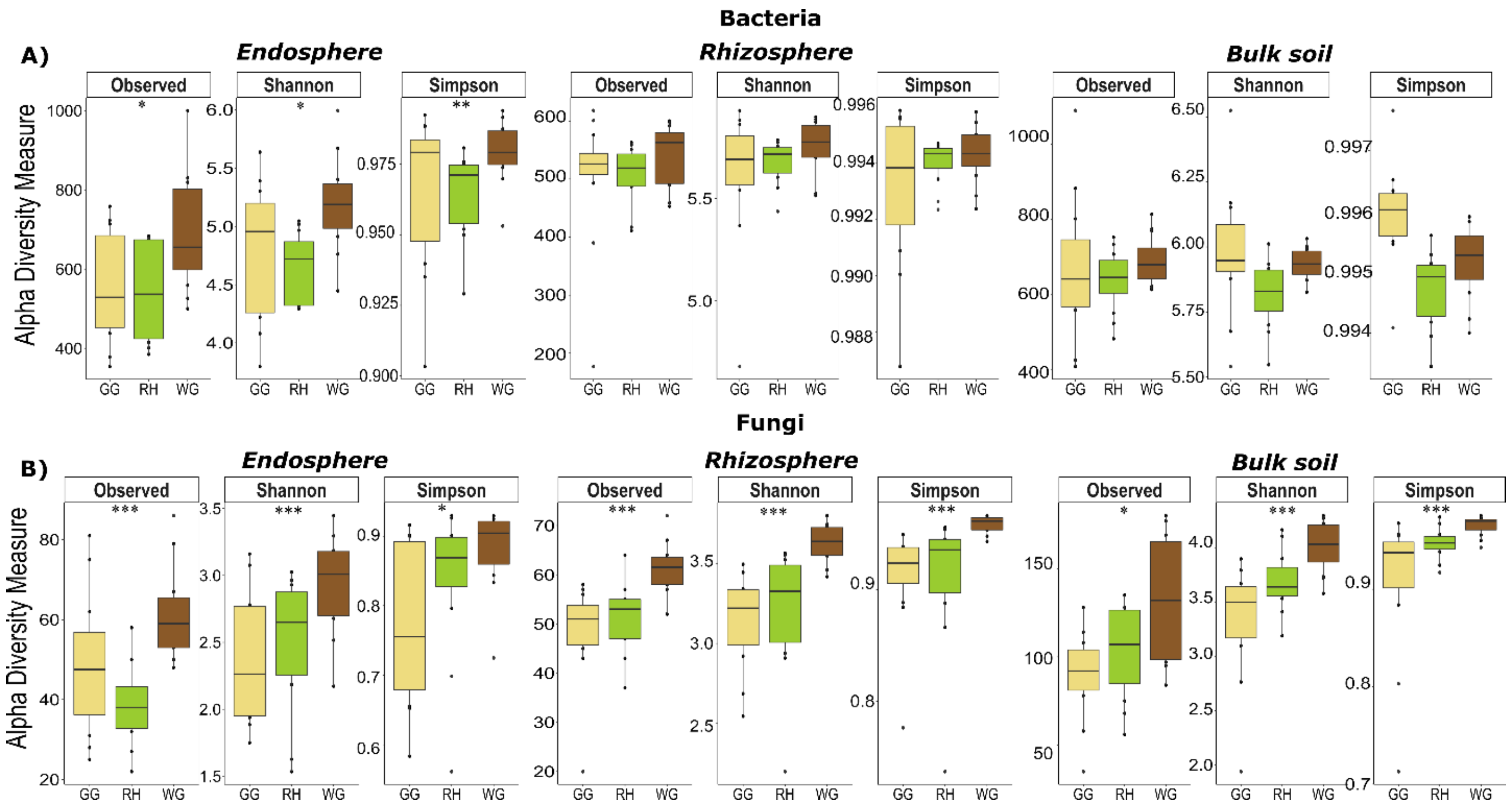

Figure S1. Observed richness and diversity (Shannon's index and Simpson indexes) of bacterial (A) and fungal (B) microbiota of different habitats (root endosphere, rhizosphere, bulk soil) between locations: yellow – Gross-Gerau (GG), green – Rauischholzhausen (RH), and brown – Weilburger Grenze (WG). Box plots show the range of variation in the median values (black lines in the middle), and the dots indicate outliers. Asterisks indicate significant differences between locations (*** = $p < 0.001$; ** = $p < 0.01$; * = $p < 0.05$).

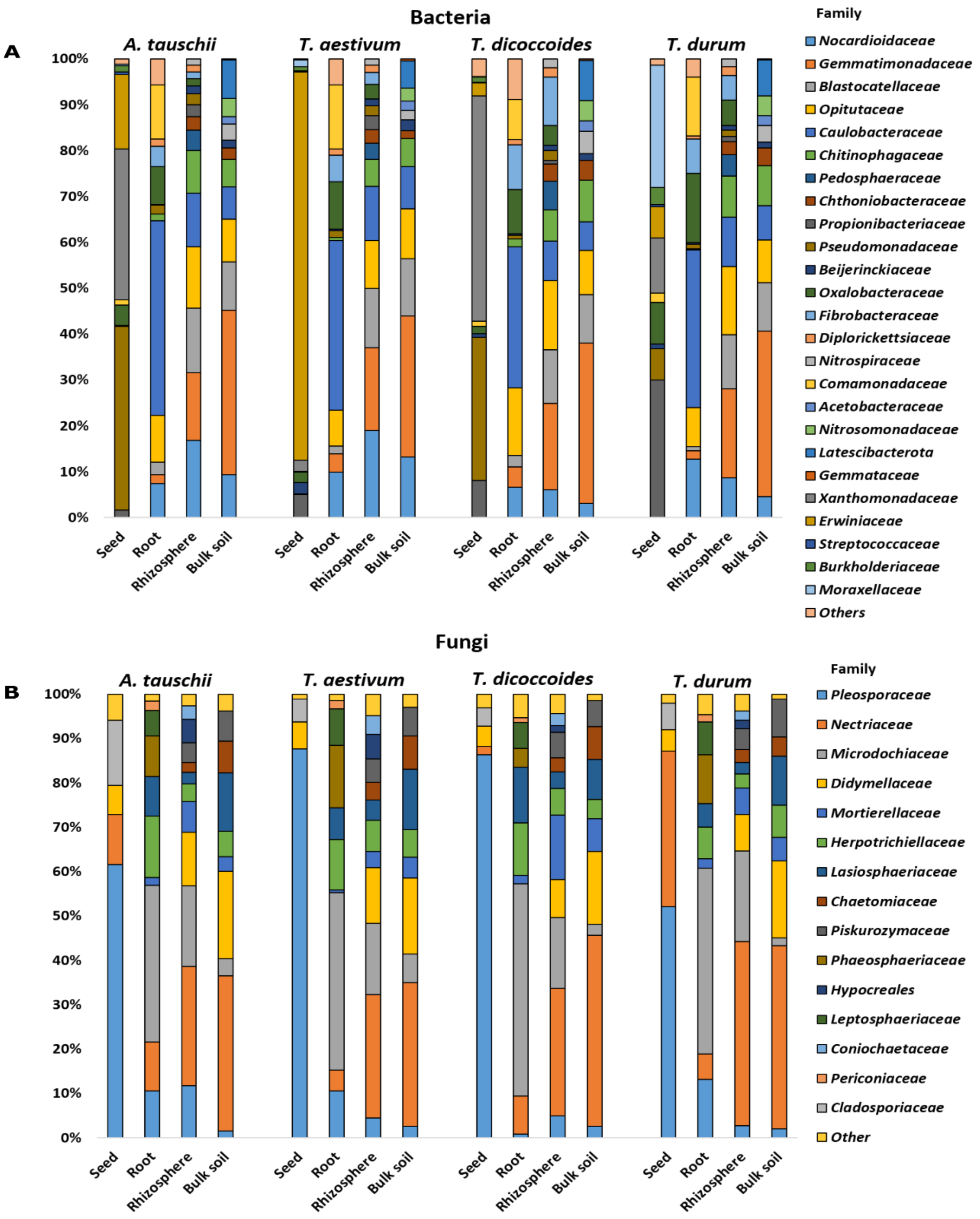

Figure S2. Bacterial (A) and fungal (B) taxa distribution in different compartments of wheat cultivars grown in three locations (GG, WG, RH). Relative abundance of 95% predominant bacterial and fungal phyla and 99% predominant genera. Each bar is the average of the data from nine samples, three per location.

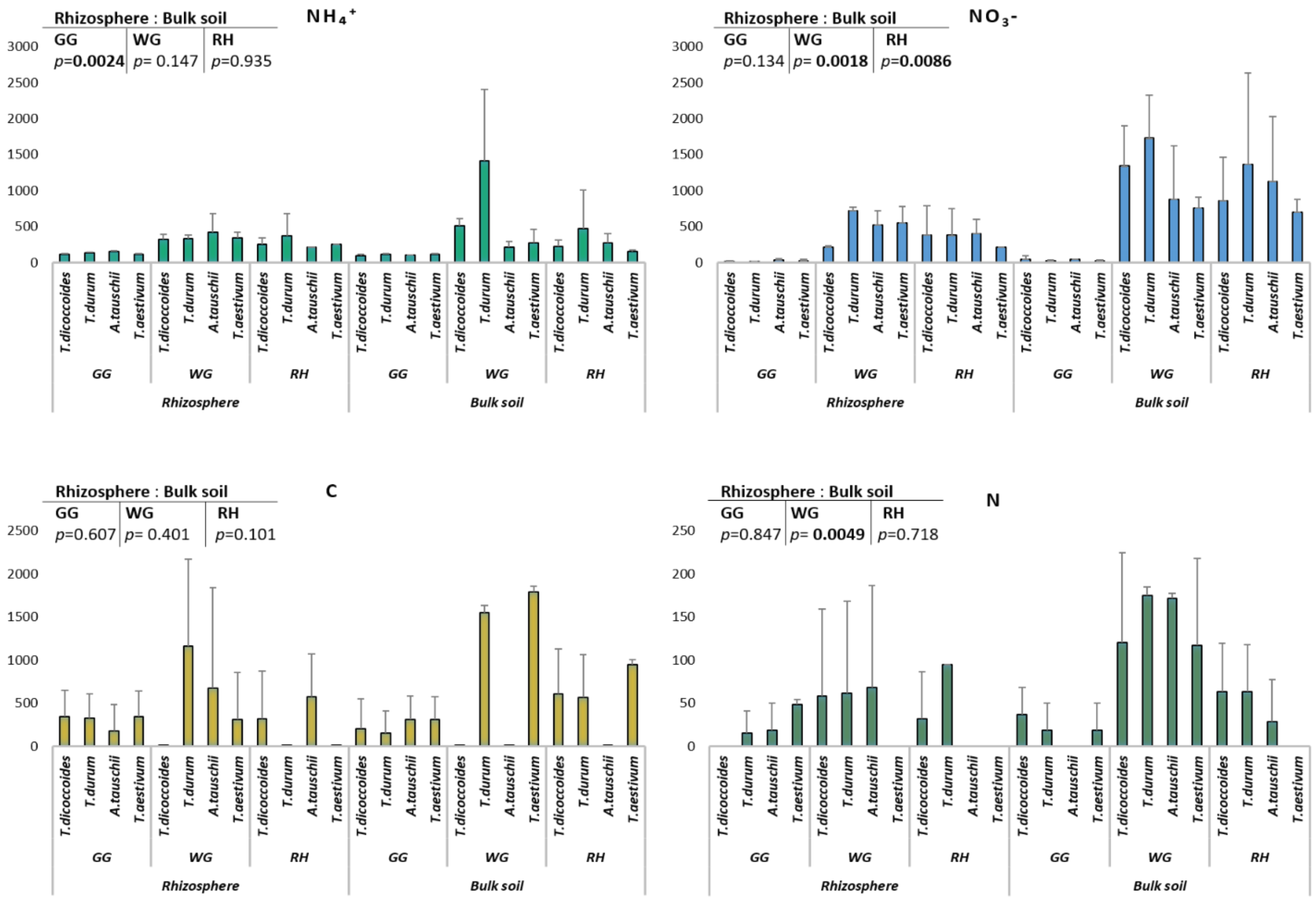

Figure S3. Soil properties. Ammonium (NH_4^+), nitrate (NO_3^-), nitrogen (N), and carbon (C) of rhizosphere and bulk soil (n=12). Soil samples collected from three locations Weilburger Grenze (WG), Groß-Gerau (GG), and Rauischholzhausen (RH). One-way ANOVA results. All parameters (NO_3^-, $p < 0.001$, NH_4^+, $p = 0.0195$, N, $p < 0.001$) except Carbon ($p = 0.0632$), significantly differed between locations (n=12). The bars represent the mean values of 3 replicates of each wheat species, error bars show standard deviation. Significance values between rhizosphere and bulk soil in each location are represented on the left corner of each barplot.

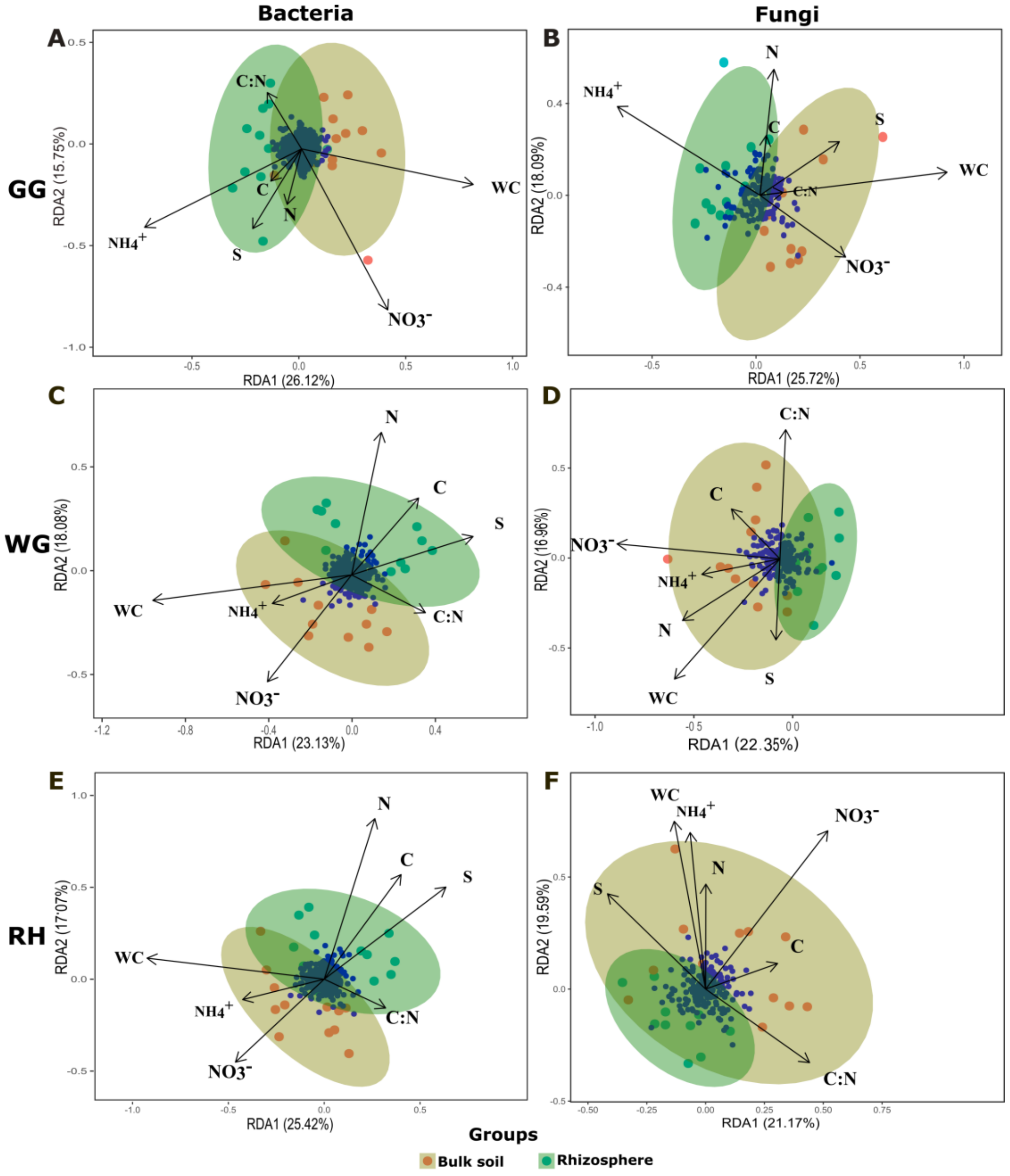

Figure S4. Constrained (canonical) ordination analyses. The effect of environmental variables on bacterial (A,C, E) and fungal (B,D,F) species in the rhizosphere and bulk soil samples of wheat that were grown in three locations: A, B) Groß-Gerau, C, D) Weilburger Grenze, and E, F) Rauischholzhausen. Green and orange dots indicate rhizosphere and bulk soil samples, respectively. Dark blue dots indicate bacterial and fungal genera.

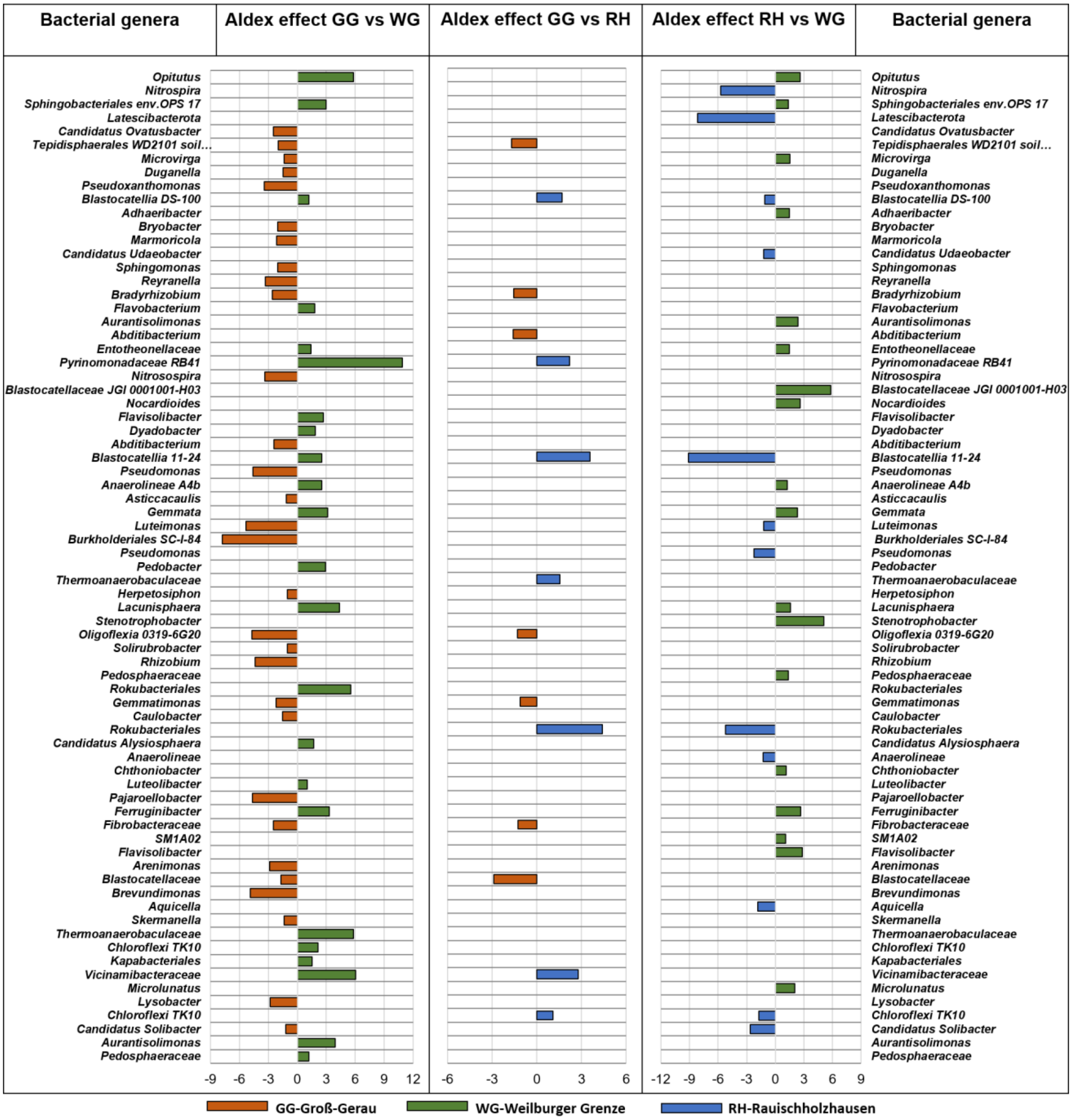

Figure S5. The bacterial structure variation between locations (GG-Groß-Gerau, WG-Weilburger Grenze, RH-Rauischholzhausen) based on differential abundance analysis of core microbiome bacterial genera of rhizosphere (*A. tauschii*). The significantly prevalent genera were identified by looking at aldex effect size. The differently abundant genera are considered as significant absolute aldex affect size bigger than 1 or lower than -1.

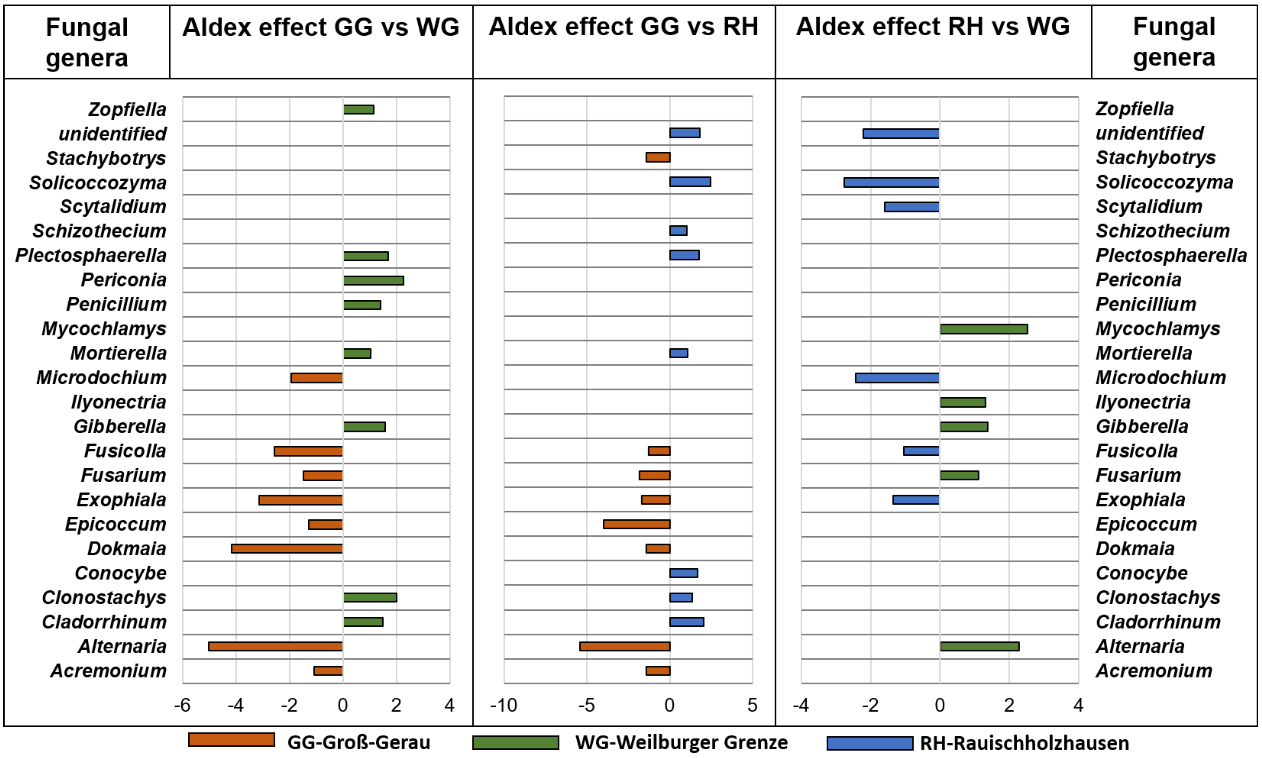

Figure S6. The fungal structure variation between locations (GG-Groß-Gerau, WG-Weilburger Grenze, RH-Rauischholzhausen) based on differential abundance analysis of core microbiome bacterial genera of rhizosphere (*A. tauschii*). The significantly prevalent genera were identified by looking at aldex effect size. The differently abundant genera are considered as significant absolute aldex affect size bigger than 1 or lower than -1.
